# Effectiveness of beetroot juice on aerobic and anaerobic exercise performance: systematic review and meta-analysis

**DOI:** 10.3389/fnut.2026.1844096

**Published:** 2026-06-29

**Authors:** Chengcheng Cai, Peijun Huang, Xiaolei Cheng, Jinghui Zhou

**Affiliations:** 1 College of Profession Tennis, Wuhan City Polytechnic, Wuhan, China; 2 School of Physical Education, Wuhan University of Science and Technology, Wuhan, China

**Keywords:** beetroot juice, high-intensity interval sprint, maximal oxygen uptake, mean power output, meta-analysis

## Abstract

**Purpose:**

Beetroot juice (BJ), a natural source of dietary nitrate, has been widely studied for its potential ergogenic effects; however, evidence integrating both aerobic and anaerobic performance outcomes remains limited. This meta-analysis evaluated the effects of BJ supplementation on maximal oxygen uptake (VO₂max), high-intensity interval sprint (HIS) performance, and mean power output (MPO).

**Methods:**

A comprehensive literature search was conducted across six databases (Wiley Online Library, Web of Science, Scopus, SpringerLink, PubMed, and ScienceDirect), supplemented by manual searches of Google Scholar. Meta-analysis, subgroup analysis, meta-regression analysis, publication bias assessment, and sensitivity analyses were performed using Stata 17.

**Results:**

Thirty-three studies were included in the review. BJ supplementation significantly improved HIS (SMD = 0.38, 95% CI 0.17 to 0.59, *p* < 0.001), MPO (SMD = 0.43, 95% CI 0.21 to 0.64, *p* < 0.001), and VO₂max (SMD = 0.30, 95% CI 0.05 to 0.55, *p* < 0.001). Heterogeneity was low across all outcomes (HIS: *I*^2^ = 11.0%, *p* = 0.343; MPO: *I*^2^ = 0.0%, *p* = 0.824; VO₂max: *I*^2^ = 0.0%, *p* = 0.970). In addition, although subgroup difference tests did not show statistically significant differences, the observed data suggest that athlete status, sport type, supplementation strategy, juice brand, and timing of intake may act as potential effect modifiers. These findings should be interpreted as hypothesis-generating and require confirmation in future studies.

**Conclusion:**

As a natural ergogenic aid, beetroot juice effectively improves both aerobic and anaerobic exercise performance and can be recommended.

**Systematic review registration:**

Identifier CRD42025636836.

## 1 Introduction

Athletic performance relies on the integrated functioning of multiple physiological systems, with aerobic and anaerobic capacity forming the crucial foundation for determining sporting achievements (1). For competitive athletes and individuals engaged in regular training, the body’s capacity for sustained energy supply, repeated high-intensity exercise, and maintaining muscular power output are all closely linked to athletic performance (2, 3). Consequently, maximal oxygen uptake, high-intensity interval sprint performance, and mean power output are widely used to assess aerobic and anaerobic exercise capacity and are common key outcome measures in sports nutrition intervention studies (1).

In recent years, nutritional supplementation strategies have garnered significant attention as important non-pharmacological means of enhancing athletic performance. Among numerous sports nutrition supplements, beetroot juice (BJ) has emerged as a research focus due to its high content of inorganic nitrate (NO₃^−^), being recognized as a natural supplement with potential performance-enhancing effects (4, 5). Following consumption, the nitrates in beetroot juice can be converted into nitric oxide (NO) via the nitrate–nitrite–nitric oxide pathway (NO₃^−^–NO₂^−^–NO) (6). NO, as a crucial signaling molecule, plays a significant role in regulating blood flow perfusion, enhancing mitochondrial function, improving muscle contraction efficiency, and reducing exercise oxygen consumption (7, 8). Consequently, beetroot juice is thought to improve athletic performance by increasing oxygen utilization efficiency, delaying the onset of fatigue, and enhancing muscle function.

Based on the aforementioned physiological mechanisms, an increasing number of studies have investigated the performance-enhancing effects of beetroot juice across various sports disciplines and populations. In the context of anaerobic exercise, researchers have primarily examined its effects on explosive power, repeated sprint capacity, and power output, including performance during high-intensity interval sprints (9–11). In the aerobic exercise domain, studies have generally focused on whether beetroot juice can enhance maximal oxygen uptake and improve endurance performance (12, 13). However, the existing evidence remains inconsistent. Some studies have reported that beetroot juice supplementation improves power output and sprint performance during high-intensity exercise and may enhance aerobic metabolic efficiency (14). In contrast, other studies found no significant effects on maximal oxygen uptake (15). These discrepancies may be attributable to differences in participation in training status, sport type, supplementation protocol (acute versus chronic), dosage, and timing of supplementation.

Moreover, current evidence on BJ supplementation has largely focused on individual aspects of athletic performance or isolated outcome measures, whereas systematic reviews and meta-analyses integrating both aerobic and anaerobic performance outcomes remain limited. Maximal oxygen uptake, high-intensity interval sprint performance, and average power output, respectively, reflect the potential effects of beetroot juice on different energy metabolism systems and dimensions of athletic performance (16–18). A systematic synthesis of evidence across these key outcomes would facilitate a more comprehensive evaluation of the ergogenic potential of beetroot juice and help clarify its practical applicability across different sporting contexts.

Therefore, this study conducted a comprehensive meta-analysis to evaluate the effects of beetroot juice supplementation on both aerobic and anaerobic exercise performance, with particular emphasis on maximal oxygen uptake, high-intensity interval sprint performance, and average power output. By synthesizing evidence from randomized controlled trials, this study aims to provide a more robust and systematic assessment of the efficacy of beetroot juice as an ergogenic aid to enhance athletic performance. The findings may also offer valuable insights for athletes, coaches, and researchers, thereby contributing to both the theoretical understanding and practical application of beetroot juice supplementation in sports performance.

## 2 Method

### 2.1 Search strategy

This study was conducted and reported in accordance with the PRISMA guidelines, and the protocol was registered with PROSPERO (CRD42025636836).

The literature search was restricted to English-language, peer-reviewed journal articles indexed in six databases: Scopus, Web of Science, PubMed, SpringerLink, Wiley Online Library, and ScienceDirect. In addition, Google Scholar was manually searched to identify supplementary literature not captured by the primary database search. The search covers the period from database inception to 1 March 2026, using the following search string: (“Beetroot Juice”) AND (“Sports Performance” OR “Athletic Performance” OR “Physical Performance” OR “Exercise Performance”) across all fields. All retrieved records were imported into EndNote for reference management and screening.

### 2.2 Inclusion and exclusion criteria

The inclusion criteria were as follows: (1) articles published in English language in peer-reviewed journals; (2) experimental studies involving human participants; (3) studies that compared Beetroot juice supplementation with a placebo condition; (4) studies reporting at least one of the following outcomes: maximal oxygen uptake, mean power output, or high-intensity interval sprint performance; and (5) studies with accessible full texts and sufficient quantitative data, including sample size, mean values, and SD, to enable data extraction and analysis.

The exclusion criteria were as follows: (1) articles not published in the English language; (2) studies using a non-experimental research design; (3) studies that did not include a placebo comparison group; (4) studies that did not report maximal oxygen uptake, mean power output, or high-intensity interval sprint performance as outcome measures; and (5) studies with inaccessible full texts or insufficient data for extraction and analysis.

### 2.3 Screening and data extraction

After importing all retrieved records into Endnote and removing duplicates, each article’s title, abstract, and full text were reviewed against predetermined inclusion and exclusion criteria. This information included the following: author names, year of publication, geographical region, research design, sample details, and results (for delayed session testing, only the first test point was extracted). In trials with more than one experimental group, data were extracted only from groups receiving exclusive beetroot juice interventions. Two authors independently performed literature screening and data extraction; any disagreements were resolved through consensus or consultation with a third author.

### 2.4 Quality assessment

Considering the potential constraints of the included articles, the Cochrane Risk of Bias tool in RevMan 5.4 software was used to evaluate the methodological quality of the included articles (19). The Cochrane Risk of Bias tool encompasses seven domains, with three possible ratings for each domain: unclear, high, or low risk of bias. To minimize subjective bias, two authors completed independent quality assessments, and a third author resolved any instances of disagreement.

### 2.5 Statistical analysis

Stata 17 was used to conduct all statistical analyses. First, because the included studies had varying outcome measures, the current analysis computed 95% CI and standardized mean deviation (SMD) assuming no sample size bias using Cohen’s *d*. To assess heterogeneity among the included studies, the *χ*^2^ test and *I*^2^ statistic were used. A fixed-effects model was chosen if *p* > 0.1 and *I*^2^ < 50%, indicating that heterogeneity between the included studies was acceptable; if *p* < 0.1 and *I*^2^ > 50% suggested that heterogeneity between the included studies was substantial, a random-effect model was chosen (20). Second, subgroup and differential analyses were performed to explore the efficacy of beetroot juice on sports performance. Third, publication bias was evaluated through funnel plots and Egger’s test, with a *p*-value < 0.05 indicating significant publication bias (21). If significant heterogeneity was present, sensitivity analyses were used to assess the robustness of the results (22). Finally, a GRADE assessment was conducted for the three outcomes included in this meta-analysis.

## 3 Results

### 3.1 Search results

**Figure 1:**
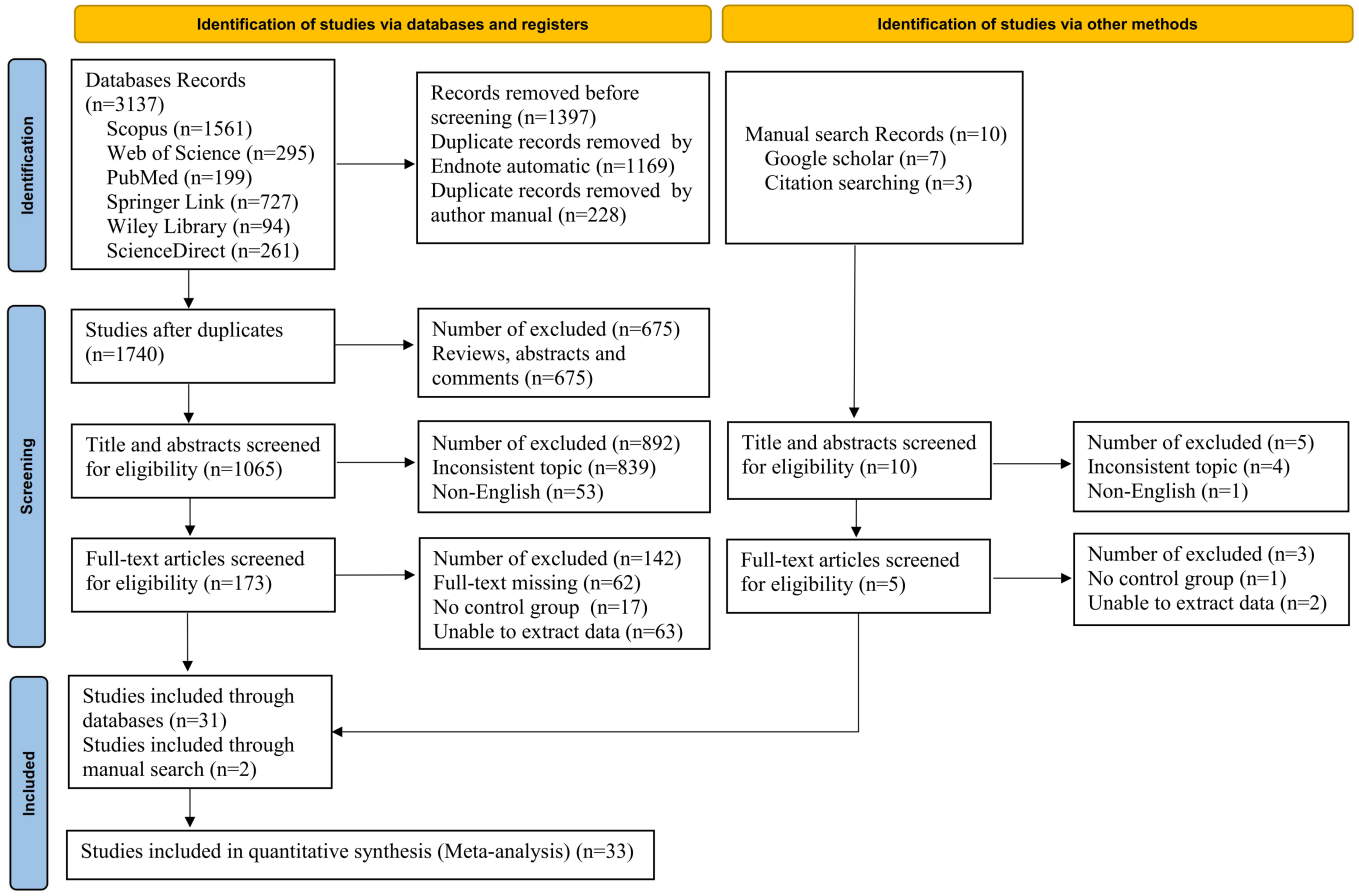
Flow diagram for included studies.

Through the database search approach, this meta-analysis identified 3,137 articles across six databases (Figure 1). A total of 1740 articles remained after duplicate articles were excluded by manual searches and automated searches using EndNote. After 675 non-peer-reviewed journal research articles were removed during the initial screening phase, following the inclusion and exclusion criteria, 892 and 142 articles were excluded, and 31 articles were included in the database search. Furthermore, to avoid overlooking any pertinent literature, a manual search was conducted using Google Scholar. Of the 10 articles retrieved via this method, 8 articles were excluded using the same screening process as the database search, and 2 articles were included. Finally, 33 articles were included in this meta-analysis.

### 3.2 Description of studies

Table 1 contains information on all 33 included studies. Participants were either professional or amateur athletes, for a total of 519. Most studies conducted through the randomized crossover controlled trial (RCCT) design reported maximum oxygen uptake, mean power output, or high-intensity interval sprint performance. The brand of beetroot juice used in most studies was Beet It from the United Kingdom, and a small number of studies used self-made beetroot juice. Intake patterns are classified as acute or chronic, with the final intake being 2 to 3 h before testing in all studies.

**Table 1:** Characteristics of studies included in the meta-analysis.

Author (year)	District	Participants	Study design	Intake method	Intake dose	B. J. brand	Last time	Outcome
Adji, Sofro (12)	Indonesia	Professional team athletes EG = 8/CG = 8 (Age: 15.8 ± 0.9)	RCT design CG: Placebo	Chronic (13d)	13*250 mL BJ (13*8.7 mmol NO_3_^−^)	Self-made	2.5 h	Maximum oxygen uptake
Antonieto, Alves do Santos (13)	Brazil	Professional individual athletes EG = 12/CG = 12 (Age: 26.8 ± 8.8)	RCCT design CG: Placebo	Acute	1 g BJ extract (No reported)	Self-made	2 h	Maximum oxygen uptake
Balsalobre-Fernández, Romero-Moraleda (30)	Spain	Professional individual athletes EG = 6/CG = 6 (Age: 26.3 ± 5.1)	RCT design CG: Placebo	Chronic (15d)	15*70 mL BJ (15*6.5 mmol NO_3_^−^)	Beet It	2.5 h	Maximum oxygen uptake
Berjisian, McGawley (9)	Iran	Professional team athletes EG = 16/CG = 16 (Age: 19.8 ± 2.2)	RCCT design CG: Placebo	Acute	60 mL BJ (6.4 mmol NO_3_^−^)	Red Beet	2.5 h	High-intensity interval sprint (Yo-Yo IR1)
Bernardi, Schoenfeld (10)	Brazil	Professional individual athletes EG = 10/CG = 10 (Age: 24.9 ± 4.6)	RCCT design CG: Placebo	Acute	400 mL BJ (9.3 mmol NO_3_^−^)	Self-made	2 h	Mean power output
Cuenca, Jodra (31)	Spain	Amateur individual athletes EG = 18/CG = 18 (Age: 22.4 ± 1.6)	RCCT design CG: Placebo	Acute	70 mL BJ (6.4 mmol NO_3_^−^)	Beet It	3 h	Mean power output
Daneshparvar., Hemmatinafar (32)	Iran	Amateur individual athletes EG = 9/CG = 9 (Age: 37 ± 7)	RCT design CG: Placebo	Acute	70 mL BJ (6.5 mmol NO_3_^−^)	Beet It	2.5 h	Maximum oxygen uptake
de Castro, de Assis Manoel (33)	Brazil	Amateur individual athletes EG = 13/CG = 13 (Age: 28.2 ± 3.0)	RCCT design CG: Placebo	Chronic (3d)	3*420 mL BJ (3*8.4 mmol NO_3_^−^)	Self-made	2 h	Maximum oxygen uptake
Domínguez, Garnacho-Castaño (14)	Spain	Professional individual athletes EG = 15/CG = 15 (Age: 21.5 ± 1.7)	RCCT design CG: Placebo	Acute	70 mL BJ (4.1 mmol NO_3_^−^)	Beet It	3 h	Mean power output
Eroglu, Kose (34)	Turkey	Professional team athletes EG = 16/CG = 16 (Age: 18.2 ± 0.4)	RCCT design CG: Placebo	Acute	140 mL BJ (12.8 mmol NO_3_^−^)	Beet It	2.5 h	Mean power output
Esen, Domínguez (11)	UK	Amateur individual athletes EG = 12/CG = 12 (Age: 21 ± 10)	RCCT design CG: Placebo	Acute	140 mL BJ (12.8 mmol NO_3_^−^)	Beet It	3 h	High-intensity interval sprint (Yo-Yo IR1)
Esen, Karayigit (35)	UK	Professional team athletes EG = 12/CG = 12 (Age: 34.7 ± 7.5)	RCCT design CG: Placebo	Acute	140 mL BJ (12.8 mmol NO_3_^−^)	Beet It	3 h	High-intensity interval sprint (Yo-Yo IR1)
Garnacho-Castaño, Pleguezuelos-Cobo (36)	Spain	Professional individual athletes EG = 10/CG = 10 (Age: 36.6 ± 4.9)	RCCT design CG: Placebo	Acute	140 ml BJ (13 mmol NO_3_^−^)	Beet It	3 h	Maximum oxygen uptake
Giv, Aminaei (37)	Iran	Amateur team athletes EG = 10/CG = 10 (Age: 21–27)	RCT design CG: Placebo	Chronic (56d)	24*100 mL BJ (24*300 mg NO_3_^−^)	Self-made	2 h	Mean power output
Jonvik, Hoogervorst (38)	Netherlands	Amateur individual athletes EG = 15/CG = 15 (Age: 18–40)	RCCT design CG: Placebo	Chronic (6d)	6*140 ml BJ (6*8.4 mmol NO_3_^−^)	Beet It	3 h	Mean power output
Kokkinoplitis and Chester (39)	UK	Amateur individual athletes EG = 18/CG = 18 (Age: 22.4 ± 1.6)	RCCT design CG: Placebo	Acute	70 ml BJ (4.1 mmol NO_3_^−^)	Beet It	3 h	Mean power output
Lansley, Winyard (40)	UK	Amateur individual athletes EG = 9/CG = 9 (Age: 21 ± 4)	RCCT design CG: Placebo	Acute	500 mL BJ (6.2 mmol NO₃^−^)	Beet It	2.5 h	Mean power output
Montalvo-Alonso, del Val-Manzano (41)	Spain	Amateur individual athletes EG = 13/CG = 13 (Age: 23.8 ± 4.9)	RCCT design CG: Placebo	Acute	70 mL BJ (6.5 mmol NO_3_^−^)	Beet It	3 h	Mean power output
Neteca, Veseta (42)	Latvia	Professional individual athletes EG = 9/CG = 9 (Age: 22.9 ± 5.6)	RCT design CG: Placebo	Acute	150 mL BJ (16.2 mmol NO_3_^−^)	Self-made	2.5 h	Maximum oxygen uptake
Nyakayiru, Jonvik (43)	Netherlands	Amateur team athletes EG = 30/CG = 30 (Age: 23 ± 1)	RCCT design CG: Placebo	Chronic (6d)	6*140 mL BJ (6*12.9 mmol NO_3_^−^)	Beet It	2.5 h	High-intensity interval sprint (Yo-Yo IR1)
Pawlak-Chaouch, Boissière (44)	France	Professional individual athletes EG = 9/CG = 9 (Age: 21.7 ± 3.7)	RCCT design CG: Placebo	Chronic (3d)	3*500 ml BJ (3*5.5 mmol NO_3_^−^)	Pajottenlander	2 h	Mean power output
Perez, Dobson (45)	USA	Amateur individual athletes EG = 20/CG = 20 (Age: 21.8 ± 2.4)	RCCT design CG: Placebo	Chronic (7d)	7*70 mL BJ (7*6.4 mmol NO_3_^−^)	Beet It	2.5 h	Maximum oxygen uptake
Pinna, Roberto (46)	Italy	Professional individual athletes EG = 14/CG = 14 (Age: 34.7 ± 7.5)	RCCT design CG: Placebo	Chronic (6d)	6*500 mL BJ (6*5.5 mmol NO_3_^−^)	Aureli	2 h	Maximum oxygen uptake
Rodríguez-Fernández, Castillo (47)	Spain	Amateur team athletes EG = 18/CG = 18 (Age: 22.8 ± 4.9)	RCCT design CG: Placebo	Acute	140 ml BJ (8.2 mmol NO_3_^−^)	Beet It	2.5 h	Mean power output
Saleh, Dev (48)	Malaysia	Amateur individual athletes EG = 30/CG = 30 (Age: 18–23)	RCCT design CG: Placebo	Acute	25 g BJ (8.1 mmol NO_3_^−^)	BeetEssence	2 h	High-intensity interval sprint (Yo-Yo IR1)
Serrano, Victor (49)	Brazil	Professional individual athletes EG = 6/CG = 6 (Age: 33.9 ± 9.9)	RCCT design CG: Placebo	Acute	140 ml BJ (5.4 mmol NO_3_^−^)	Self-made	2 h	Maximum oxygen uptake
Tan, Merrill (50)	USA	Amateur team athletes EG = 15/CG = 15 (Age: 20 ± 1)	RCCT design CG: Placebo	Acute	2*70 ml BJ (12.0 mmol NO_3_^−^)	Beet It	2.5 h	High-intensity interval sprint (Yo-Yo IR1)
Thompson, Wylie (51)	UK	Amateur team athletes EG = 16/CG = 16 (Age: 24 ± 5)	RCCT design CG: Placebo	Chronic (7d)	7*140 mL BJ (7*12.8 mmol NO_3_^−^)	Beet It	2.5 h	Intermittent sprint test (IST)
Thompson, Vanhatalo (52)	UK	Professional team athletes EG = 36/CG = 36 (Age: 15.8 ± 0.9)	RCCT design CG: Placebo	Chronic (5d)	5*70 mL BJ (5*6.4 mmol NO_3_^−^)	Beet It	2.5 h	High-intensity interval sprint (Yo-Yo IR1)
Vitti, Bruneau Jr. (53)	USA	Amateur individual athletes EG = 17/CG = 17 (Age: 19.0 ± 1.0)	RCCT design CG: Placebo	Acute	100 ml BJ (No reported)	Sur nutrition	2 h	Maximum Oxygen Uptake
Williams, Martin (54)	USA	Amateur individual athletes EG = 11/CG = 11 (Age: 22.1 ± 2.4)	RCCT design CG: Placebo	Acute	70 ml BJ (4.1 mmol NO_3_^−^)	Beet It	2 h	Mean power output
Wylie, Mohr (55)	UK	Amateur team athletes EG = 14/CG = 14 (Age: 22 ± 2)	RCCT design CG: Placebo	Chronic (2d)	2*140 ml BJ (2*8.2 mmol NO_3_^−^)	Beet It	2.5 h	High-intensity interval sprint (Yo-Yo IR1)
Wylie, Bailey (23)	UK	Amateur team athletes EG = 10/CG = 10 (Age: 21 ± 1)	RCCT design CG: Placebo	Chronic (3d)	3*140 mL BJ (3*8.4 mmol NO_3_^−^)	Beet It	2 h	Mean power output

RCT, randomized controlled trial; RCCT, randomized crossover controlled trial.

### 3.3 Quality assessment results

**Figure 2:**
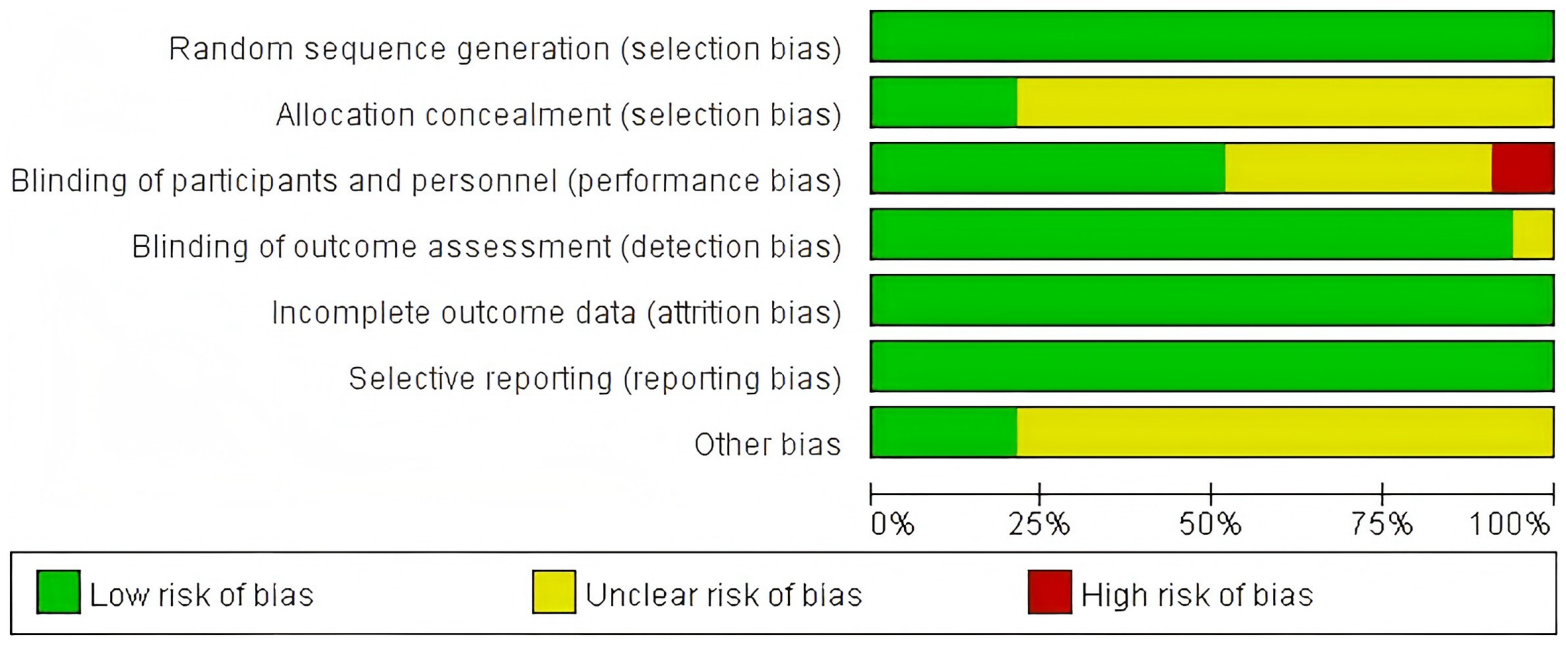
The overall risk of bias.

**Figure 3:**
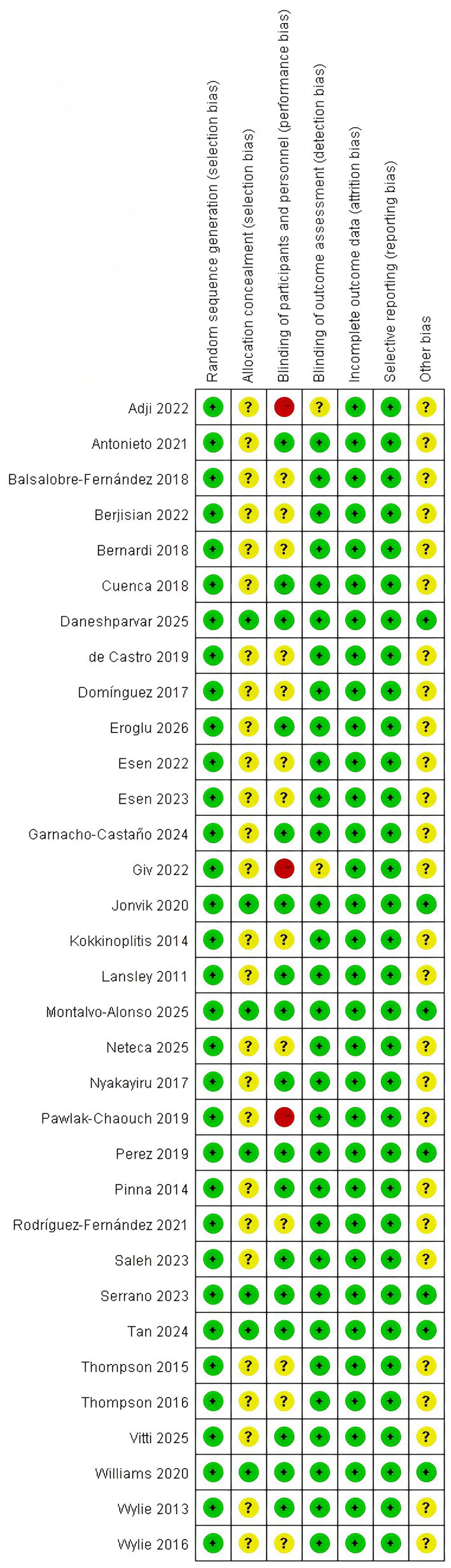
The individual risk of bias.

Figure 2 displays the overall risk-of-bias profile, whereas Figure 3 displays the risk-of-bias profile for each research separately. The results suggest that the primary potential risk stems from the specific randomization plan, which is not reported in most studies. However, apart from this, the risk of bias was generally low, and the overall quality of the included articles was deemed good.

### 3.4 Overall effectiveness results

**Figure 4:**
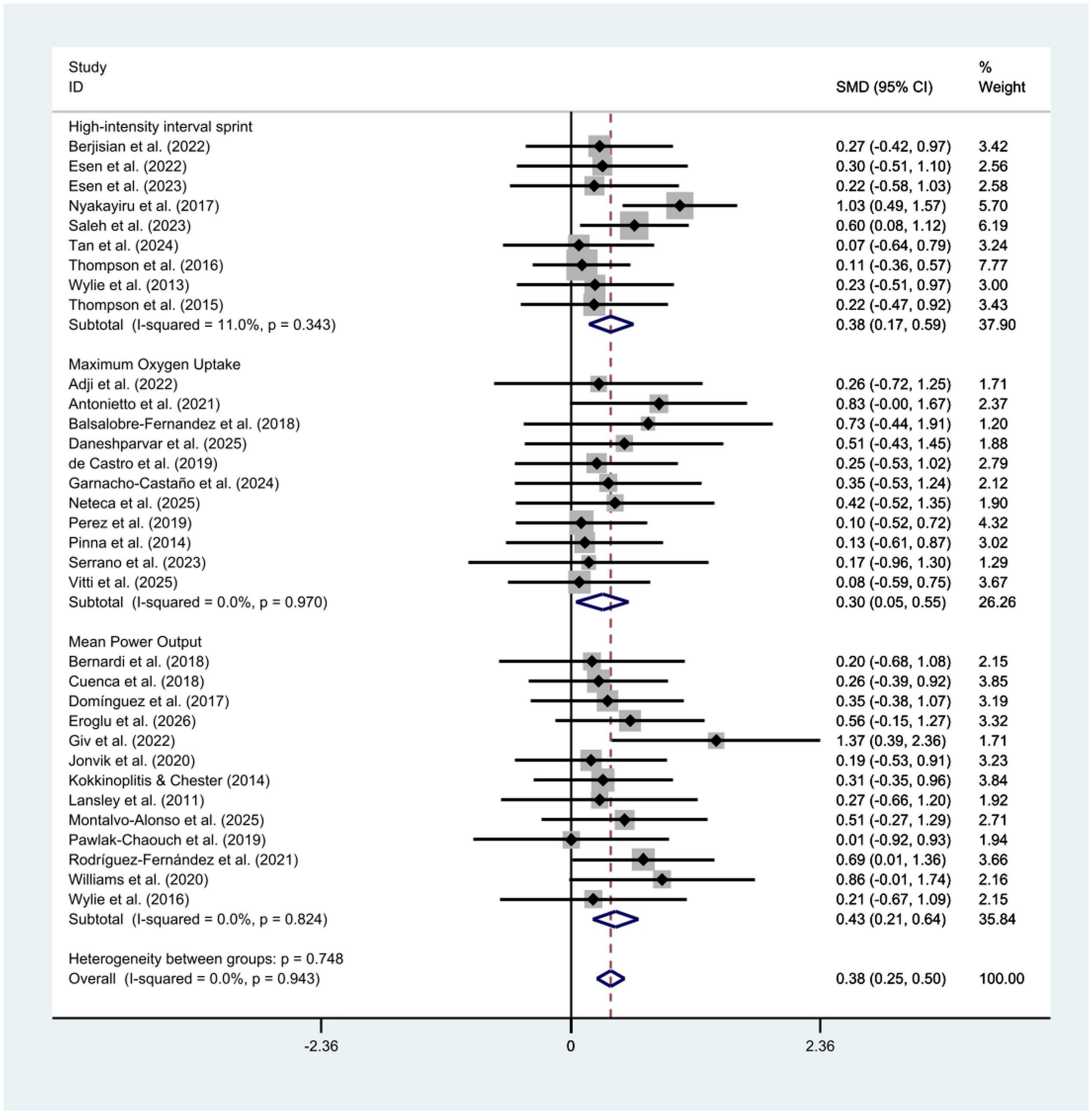
Forest Plot of the Overall Effectiveness.

This meta-analysis included 33 articles with 33 independent data points about maximum oxygen uptake, mean power output, or high-intensity interval sprint performance. As shown in Figure 4, first, 9 experiments reported high-intensity interval sprint performance as an outcome; the effect size (SMD) indicated that beetroot juice supplementation was significantly effective in enhancing high-intensity interval sprint performance (*d* = 0.38, 95% CI [0.17, 0.59], *p* < 0.001). In addition, the *χ*^2^ test showed low heterogeneity (*I*^2^ = 11.0%, *p* = 0.343), suggesting that the overall effectiveness of beetroot juice supplementation on high-intensity interval sprint performance was stable. Second, 13 experiments reported the mean output as an outcome; the effect size (SMD) indicated that beetroot juice supplementation had significant effectiveness in enhancing mean power output (*d* = 0.43, 95% CI [0.21, 0.64], *p* < 0.001). In addition, the *χ*^2^ test indicated no detectable heterogeneity (*I*^2^ = 0.0%, *p* = 0.824), suggesting that the overall effectiveness of beetroot juice supplementation on mean power output was stable. Third, 11 experiments reported maximum oxygen uptake as an outcome; the effect size (SMD) indicated that beetroot juice supplementation was significantly effective in enhancing maximum oxygen uptake (*d* = 0.30, 95% CI [0.05, 0.55], *p* < 0.001). In addition, the *χ*^2^ test indicated no detectable heterogeneity (*I*^2^ = 0.0%, *p* = 0.970), suggesting that the overall effectiveness of beetroot juice supplementation on maximum oxygen uptake was stable.

### 3.5 Results of the subgroup analysis and heterogeneity test between groups

The results of the subgroup analyses for the heterogeneity test between groups of high-intensity interval sprint performance are presented in Table 2. Stratification by geographical region showed that effect sizes were statistically significant in Europe (*d* = 0.38, *p* < 0.01) and Asia (*d* = 0.48, *p* < 0.05), but not statistically significant in North America (*d* = 0.07, *p* > 0.05). Subgroup analysis by athlete status showed statistically significant synthesis effect sizes for studies that used amateur athletes (*d* = 0.50, *p* < 0.001), but not for professional athletes (*d* = 0.17, *p* > 0.05). Subgroup analysis by type of sports showed that studies had statistically significant effect sizes for participants who were team sports athletes (*d* = 0.34, *p* < 0.01) and individual sports athletes (*d* = 0.51, *p* < 0.05). Subgroup analysis by intake method showed both statistically significant effect sizes for studies conducted on chronic intake (*d* = 0.41, *p* < 0.01) and acute intake (*d* = 0.35, *p* < 0.05). Subgroup analysis by beetroot juice brand showed both statistically significant synthesis effect sizes for studies using Beet It (*d* = 0.34, *p* < 0.01) and other brands (*d* = 0.48, *p* < 0.05). Subgroup analysis by last intake time showed that studies had statistically significant effect sizes 2 h prior (*d* = 0.60, *p* < 0.05) and 2.5 h prior (*d* = 0.35, *p* < 0.01), but not at 3 h prior (*d* = 0.26, *p* > 0.05). In addition, all between-group heterogeneities were non-significant.

**Table 2:** Subgroup analysis and heterogeneity test between groups (HIS).

Subgroup	*K*	Heterogeneity		Meta-analysis		Intergroup heterogeneity		
		*I* ^2^	*p*	*d*	95% CI	HS	df	*p*
Study design						NA	NA	NA
RCCT	9	11.0%	0.343	0.38***	[0.11, 0.59]			
RCT	0	NA	NA	NA	NA			
District						0.94	2	0.625
Europe	6	33.3%	0.186	0.38**	[0.12, 0.64]			
North America	1	NA	NA	0.07	[−0.64, 0.79]			
Asia	2	0.0%	0.457	0.48*	[0.07, 0.90]			
Athlete status						2.22	1	0.136
Professional athletes	3	0.0%	0.916	0.17	[−0.18, 0.52]			
Amateur athletes	6	24.1%	0.253	0.50***	[0.24, 0.76]			
Type of sport						0.47	1	0.494
Team sports	7	26.3%	0.228	0.34**	[0.10, 0.58]			
Individual sports	2	0.0%	0.535	0.51*	[0.08, 0.95]			
Intake method						0.09	1	0.770
Chronic intake	4	58.7%	0.064	0.41**	[0.12, 0.70]			
Acute intake	5	0.0%	0.803	0.35*	[0.04, 0.65]			
Beetroot juice brand						0.33	1	0.568
Beet It	7	26.0%	0.230	0.34**	[0.10, 0.59]			
Other brands	2	0.0%	0.457	0.48*	[0.07, 0.90]			
Last time intake						0.92	2	0.630
2 h prior	1	NA	NA	0.60*	[0.08, 1.12]			
2.5 h prior	6	37.9%	0.154	0.35**	[0.10, 0.60]			
3 h prior	2	0.0%	0.896	0.26	[−0.31, 0.83]			

*p < 0.05; **p < 0.01; ***p < 0.001; *K*, number of effect sizes; *d*, Cohen’s *d*; 95% CI, 95% confidence interval; HS, heterogeneity statistic; df, degrees of freedom.

Second, the results of the subgroup analyses of the heterogeneity test between groups of mean power output are shown in Table 3. The effect sizes for studies in the subgroup analysis by study design were statistically significant, using both RCCT (*d* = 0.38, *p* < 0.01) and RCT (*d* = 1.37, *p* < 0.01). Analysis by geographical region showed that effect sizes were statistically significant in Europe (*d* = 0.33, *p* < 0.05) and Asia (*d* = 0.84, *p* < 0.01) but not statistically significant in North America (*d* = 0.86, *p* > 0.05) or South America (*d* = 0.20, *p* > 0.05). Subgroup analysis by athlete status showed statistically significant synthesis effect sizes for studies that used participants who were amateur athletes (*d* = 0.47, *p* < 0.01), but not statistically significant for professional athletes (*d* = 0.36, *p* < 0.05). Subgroup analysis by type of sports showed studies had statistically significant effect sizes that were used by participants, who were team sports athletes (*d* = 0.66, *p* < 0.01), and individual sports athletes (*d* = 0.32, *p* < 0.05). Subgroup analysis by intake method showed both statistically significant effect sizes for studies conducted in acute intake (*d* = 0.44, *p* < 0.01) but not significant for chronic intake (*d* = 0.38, *p* > 0.05). Subgroup analysis by beetroot juice brand showed both statistically significant synthesis effect sizes for studies using beet It (*d* = 0.42, *p* < 0.01) and self-made (*d* = 0.72, *p* < 0.05), but not significant in other brands (*d* = 0.01, *p* > 0.05). Subgroup analysis by last intake time showed studies had statistically significant effect sizes with 2 h prior (*d* = 0.51, *p* < 0.05), 2.5 h prior (*d* = 0.55, *p* < 0.05), but not statistically significant in 3 h prior (*d* = 0.31, *p* > 0.05). In addition, all between-group heterogeneities were non-significant.

**Table 3:** Subgroup analysis and heterogeneity test between groups (MPO).

Subgroup	*K*	Heterogeneity		Meta-analysis		Intergroup heterogeneity		
		*I* ^2^	*p*	*d*	95% CI	HS	df	*p*
Study design						3.71	1	0.053
RCCT	12	0.0%	0.977	0.38**	[0.16, 0.60]			
RCT	1	NA	NA	1.37**	[0.39, 2.36]			
District						3.73	3	0.292
Europe	9	0.0%	0.980	0.33*	[0.08, 0.58]			
North America	1	NA	NA	0.86	[−0.01, 1.74]			
South America	1	NA	NA	0.20	[−0.68, 1.08]			
Asia	2	42.2%	0.188	0.84**	[0.26, 1.41]			
Athlete status						0.22	1	0.638
Professional athletes	5	0.0%	0.887	0.36*	[0.01, 0.71]			
Amateur athletes	8	0.0%	0.526	0.47**	[0.19, 0.74]			
Type of sport						2.00	1	0.157
Team sports	4	3.1%	0.377	0.66**	[0.27, 1.05]			
Individual sports	9	0.0%	0.967	0.32*	[0.07, 0.58]			
Intake method						0.06	1	0.806
Chronic intake	4	39.4%	0.175	0.38	[−0.05, 0.81]			
Acute intake	9	0.0%	0.963	0.44**	[0.19, 0.69]			
Beetroot Juice brand						1.58	2	0.454
Beet It	10	0.0%	0.970	0.42**	[0.18, 0.65]			
Other brands	1	NA	NA	0.01	[−0.92, 0.93]			
Self-made	2	67.1%	0.081	0.72*	[0.07, 1.38]			
Last time intake						0.95	2	0.621
2 h prior	5	29.1%	0.227	0.51*	[0.10, 0.91]			
2.5 h prior	3	0.0%	0.780	0.55*	[0.12, 0.98]			
3 h prior	5	0.0%	0.984	0.31	[−0.01, 0.63]			

*p < 0.05; **p < 0.01; ***p < 0.001; *K*, number of effect sizes; *d*, Cohen’s d; 95% CI, 95% confidence interval; HS, heterogeneity statistic; df, degrees of freedom.

Third, the results of the subgroup analysis heterogeneity test between groups of mean power output are shown in Table 4. Subgroup analysis by athlete status showed statistically significant synthesis effect sizes for studies that used participants who were professional athletes (*d* = 0.41, *p* < 0.05) but not statistically significantly used amateur athletes (*d* = 0.17, *p* > 0.05). Subgroup analysis by type of sport showed that studies had statistically significant effect sizes for participants who were individual sports athletes (*d* = 0.30, *p* < 0.01) and team sports athletes (*d* = 0.31, *p* > 0.05). Subgroup analysis by intake method showed a statistically significant effect size for studies conducted on acute intake (*d* = 0.38, *p* < 0.05) but not significant for chronic intake (*d* = 0.22, *p* > 0.05). Subgroup analysis by beetroot juice brand showed statistically significant synthesis effect sizes for studies using self-made (*d* = 0.41, *p* < 0.05) but not significant for Beet It (*d* = 0.32, *p* > 0.05) and other brands (*d* = 0.10, *p* > 0.05). All other subgroups were not significant. In addition, all between-group heterogeneities were not significant.

**Table 4:** Subgroup analysis and heterogeneity test between groups (VO₂max).

Subgroup	*K*	Heterogeneity		Meta-analysis		Intergroup heterogeneity		
		*I* ^2^	*p*	*d*	95% CI	HS	df	*p*
Study design						0.54	1	0.462
RCCT	7	0.0%	0.869	0.24	[−0.05, 0.53]			
RCT	4	0.0%	0.946	0.46	[−0.04, 0.96]			
District						1.22	3	0.747
Europe	4	0.0%	0.860	0.34	[−0.11, 0.79]			
North America	2	0.0%	0.969	0.09	[−0.36, 0.55]			
South America	3	0.0%	0.520	0.45	[−0.06, 0.95]			
Asia	2	0.0%	0.727	0.39	[−0.29, 1.07]			
Athlete status						0.87	1	0.352
Professional athletes	7	0.0%	0.908	0.41*	[0.06, 0.76]			
Amateur athletes	4	0.0%	0.935	0.17	[−0.19, 0.54]			
Type of sport						0.00	1	0.960
Team sports	2	0.0%	0.893	0.31	[−0.34, 0.97]			
Individual sports	9	0.0%	0.907	0.30*	[0.02, 0.57]			
Intake method						0.39	1	0.535
Chronic intake	5	0.0%	0.919	0.22	[−0.14, 0.58]			
Acute intake	6	0.0%	0.836	0.38*	[0.02, 0.73]			
Beetroot juice brand						0.88	2	0.644
Beet It	4	0.0%	0.775	0.32	[−0.10, 0.73]			
Other brands	2	0.0%	0.922	0.10	[−0.39, 0.60]			
Self-made	5	0.0%	0.842	0.41*	[0.01, 0.82]			
Last time intake						0.05	0	0.977
2 h prior	5	0.0%	0.698	0.27	[−0.08, 0.63]			
2.5 h prior	5	0.0%	0.885	0.32	[−0.07, 0.71]			
3 h prior	1	NA	NA	0.35	[−0.53, 1.24]			

*p < 0.05; **p < 0.01; ***p < 0.001; *K*, number of effect sizes; *d*, Cohen’s d; 95% CI, 95% confidence interval; HS, heterogeneity statistic; df, degrees of freedom.

### 3.6 Results of publication bias and sensitivity analysis

**Figure 5:**
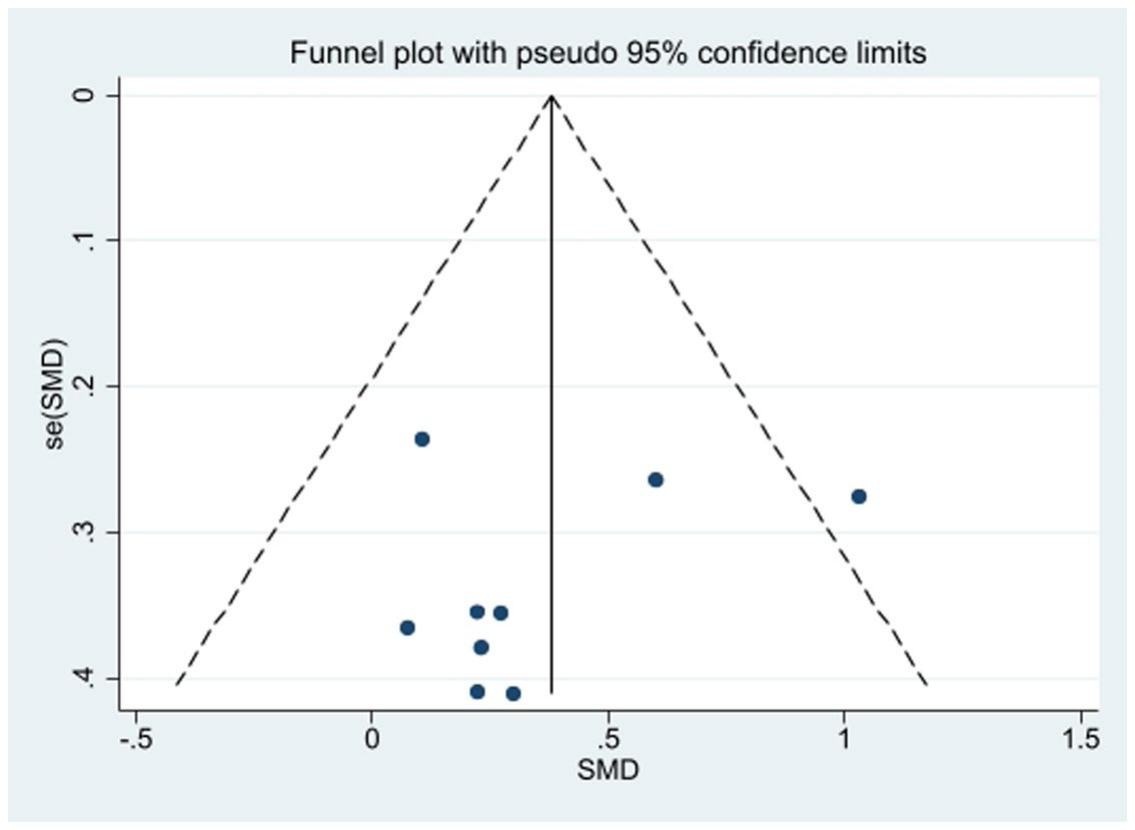
Funnel plot of publication bias of HIS.

**Figure 6:**
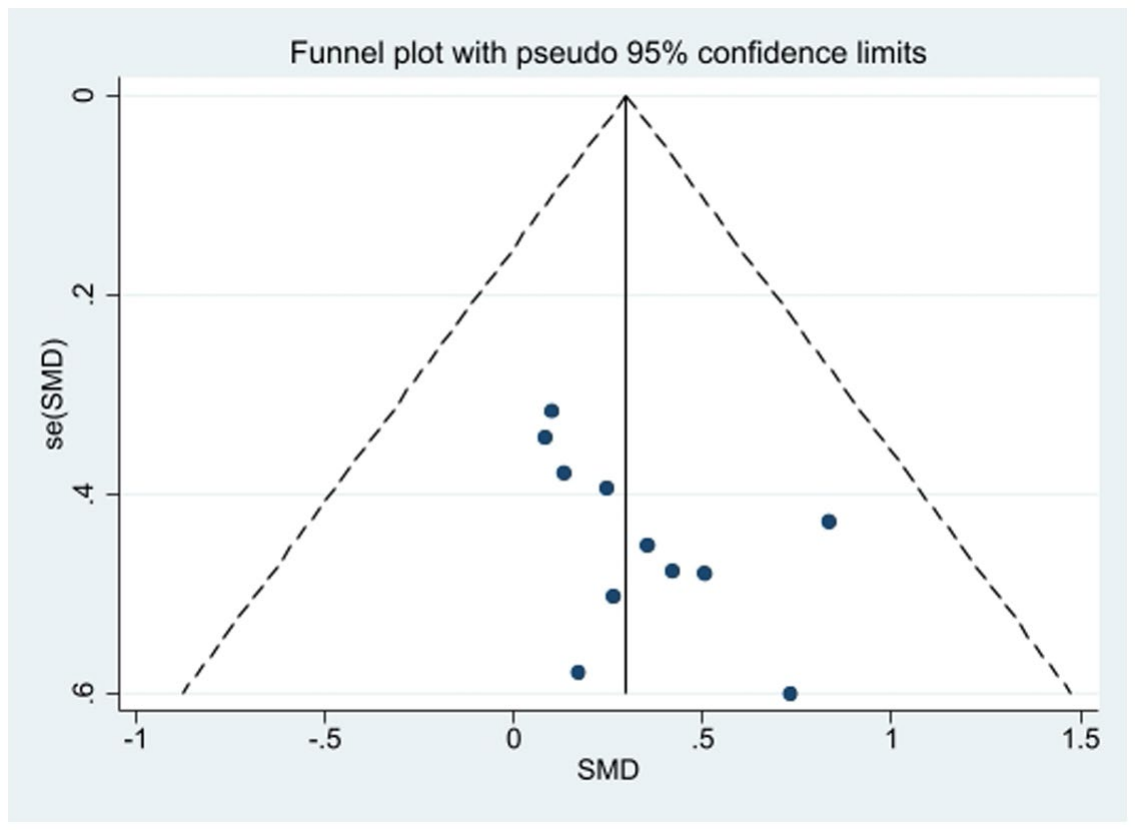
Funnel plot of VO2MAX.

**Figure 7:**
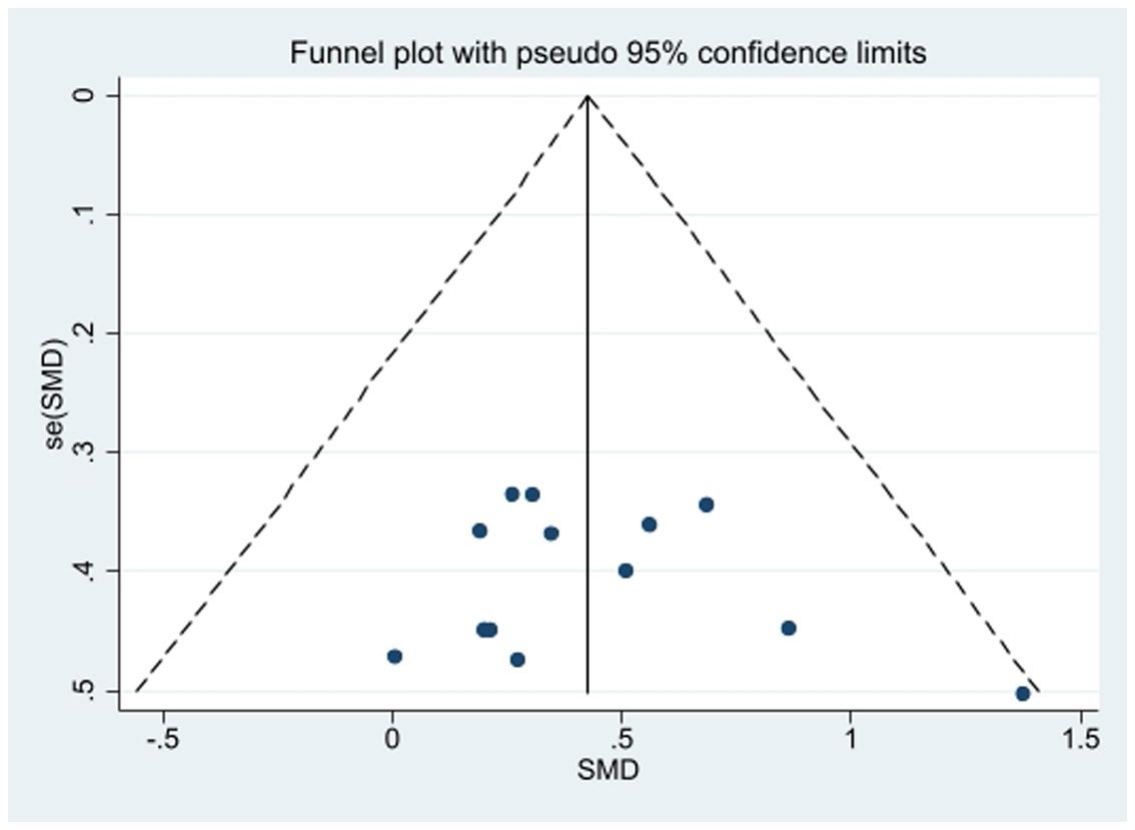
Funnel plot of publication bias of MPO.

The funnel plots were not unduly asymmetrical (Figures 5–7). Notwithstanding the funnel plot, limited sample size or design problems might indicate a tiny possible publication bias, which could result in inflated study findings. To further assess publication bias, the data were evaluated using Egger’s test, which revealed that the *p*-values (P_HIS_ = 0.452; P_MPO_ = 506; P_VO₂max_ = 0.070) were more than 0.05. Taken together, the funnel plots and Egger’s test results show that the study’s dependability was rather high and that there was no discernible publication bias. In addition, as the heterogeneity across all three meta-analyses was low or non-existent, sensitivity analyses were not required.

### 3.7 Results of the GRADE assessment of the certainty of evidence

As shown in Table 5, the GRADE assessment indicated a moderate certainty of evidence for HIS, MPO, and VO_2_max. All three outcomes were downgraded by one level due to risk-of-bias concerns, mainly due to unclear reporting of randomization and allocation concealment, and potential blinding issues related to the distinctive color and taste of beetroot juice. No further downgrading was applied for inconsistency, indirectness, imprecision, or publication bias.

**Table 5:** Results of GRADE assessment.

Outcome	Risk of bias	Inconsistency	Indirectness	Imprecision	Publication bias	Certainty
HIS	Serious^1^	Not serious^2^	Not serious	Not serious^3^	Not serious^4^	Moderate
MPO	Serious^1^	Not serious^2^	Not serious	Not serious^3^	Not serious^4^	Moderate
VO₂max	Serious^1^	Not serious^2^	Not serious	Not serious^3^	Not serious^4^	Moderate

1 Downgraded one level for risk of bias due to unclear reporting of randomization/allocation concealment and potential blinding concerns related to the distinctive color and taste of beetroot juice.

2 Not downgraded for inconsistency because heterogeneity was low across outcomes: HIS, *I*^2^ = 11.0%; MPO, *I*^2^ = 0.0%; VO₂max, *I*^2^ = 0.0%.

3 Not downgraded for imprecision because pooled effects were significant and confidence intervals did not cross the line of no effect, although individual studies generally had small sample sizes.

4 Not downgraded for publication bias because funnel plots were broadly symmetrical and Egger’s tests were not significant.

## 4 Discussion

This study systematically evaluated the effects of beetroot juice on aerobic and anaerobic exercise performance. The overall results indicate that beetroot juice supplementation significantly enhances high-intensity interval sprint performance, mean power output, and maximal oxygen uptake, with low overall heterogeneity suggesting robust stability in these ergogenic effects.

### 4.1 Beetroot juice enhances aerobic exercise performance

Regarding anaerobic exercise performance, this study found that beetroot juice supplementation significantly increased HIS and MPO. Both HIS and MPO reflect power output capacity and fatigue resistance during short-duration high-intensity exercise, suggesting that beetroot juice holds practical significance for improving anaerobic and high-intensity interval training performance (23, 24). The underlying physiological mechanisms likely involve dietary nitrates being metabolized into nitric oxide, thereby enhancing skeletal muscle blood perfusion, improving muscular contraction efficiency, and delaying fatigue onset (25). Therefore, for sports requiring repeated sprints or sustained high-power output, beetroot juice may represent a valuable nutritional supplementation strategy.

### 4.2 Beetroot juice enhances anaerobic exercise performance

Regarding aerobic exercise performance, this study found that beetroot juice supplementation similarly increased VO₂max, albeit with a relatively modest effect. Although the effect size was smaller than that observed for anaerobic-related metrics, it nonetheless suggests that beetroot juice exerts a positive influence on aerobic capacity. Maximum oxygen uptake is a crucial indicator of aerobic exercise capability; its improvement indicates that beetroot juice may enhance the body’s oxygen transport and utilization efficiency (26, 27). Previous research suggests that nitrates in beetroot juice may enhance endurance-related performance by improving vasodilation via the NO pathway, increasing muscle oxygen delivery, and optimizing mitochondrial energy metabolism efficiency (28). However, compared to anaerobic metrics, VO₂max is influenced by multiple factors, including training level, cardiopulmonary function, and testing methodology (29). Furthermore, while this small improvement in VO₂max may be meaningful for competitive athletes, it may be of less practical significance for recreational exercisers.

### 4.3 Factors influencing the efficacy of beetroot juice

Regarding subgroup results across different outcome measures, this meta-analysis observed descriptive differences in HIS, MPO, and VO₂max. Specifically, for high-intensity interval sprint performance (HIS), statistically significant effects were observed in several subgroups, such as amateur athletes, team and individual sports, acute and chronic supplementation, and intake 2 to 2.5 h before exercise, whereas other subgroups did not reach statistical significance. For mean power output (MPO), significant results were observed mainly in the acute supplementation subgroup, the Beet It brand subgroup, and the 2- or 2.5-h before competition intake subgroups. In addition, significant VO₂max results were mainly observed in professional athletes, individual sports participants, those receiving acute supplementation, and the self-prepared formulation subgroup. These findings suggest that the observed effect sizes may vary to some extent across different study designs, participant characteristics, sports types, and supplementation protocols.

However, it should be emphasized that the tests for between-subgroup heterogeneity in this study did not show statistically significant differences. However, although the current evidence is insufficient to demonstrate that athlete level, sport type, supplementation method, brand, or timing of intake has a true moderating effect on the efficacy of beetroot juice, these explanations remain plausible mechanistic interpretations. Therefore, the subgroup findings in this study are regarded as descriptive and hypothesis-generating and may provide a reference for future studies to further explore potential influencing factors, such as participants’ sport status, sport type, supplementation dose, brand differences, and timing of intake.

### 4.4 Limitations and future directions

First, although the overall meta-analysis showed low heterogeneity, the number of studies included in some subgroups was limited, with certain subgroups containing only one or two studies. This compromised the stability and precision of the effect estimates, thereby reducing the interpretative strength of the subgroup findings. Second, this study mainly focused on sport performance outcomes and did not investigate dose–response relationships, long-term supplementation effects, or potential physiological mechanisms. Future research is needed for validation. Third, due to the limited information reported in the original studies, it was not possible to analyze a wide range of participant-level characteristics, such as gender and age differences. Finally, the tests for between-subgroup heterogeneity for all outcome measures in this study did not show statistically significant differences, suggesting that the current evidence is insufficient to support clear beetroot juice effects across different study characteristics or supplementation conditions. Accordingly, these subgroup findings should be regarded as exploratory indications of potential influencing factors rather than definitive conclusions. Future research requires high-quality, adequately powered, and rigorously reported studies to validate the optimal application conditions of beetroot juice across different populations, sport disciplines, and supplementation protocols.

## 5 Conclusion

This meta-analysis suggests that beetroot juice supplementation can effectively improve both anaerobic and aerobic exercise performance. However, given the small-to-moderate effect sizes and the methodological diversity across the included studies, these findings should be interpreted cautiously. In addition, the subgroup results should be considered exploratory, as between-group heterogeneity tests were not statistically significant. In summary, while beetroot juice may be a useful sports nutrition strategy, its practical application should consider exercise type, dosage, timing, and athlete characteristics.

## Data Availability

The raw data supporting the conclusions of this article will be made available by the authors, without undue reservation.
